# Percutaneous edge-to-edge repair in congenital heart disease: Preliminary results of a promising new technique

**DOI:** 10.1016/j.ijcchd.2022.100370

**Published:** 2022-04-26

**Authors:** Alexandre Silini, Xavier Iriart

**Affiliations:** University Hospital of Bordeaux, Department of Pediatric and Adult Congenital Cardiology, France

**Keywords:** Systemic right ventricle, Tricuspid regurgitation, Transposition of the great arteries, Congenitally corrected, Heart left hypoplasia syndrome, Edge-to-edge repair, MitraClip, Mitral cleft

## Abstract

Percutaneous edge-to-edge repair has become a valid technique for symptomatic mitral and tricuspid regurgitation in patients with acquired cardiopathies deemed ineligible for surgery. In congenital heart disease (CHD) and more specifically in systemic right ventricle atrioventricular valve regurgitation, it has a pivotal role in the disease course, functional prognosis, systemic ventricular function, and mortality. While medical therapy is not unequivocally effective, surgery (valve repair or replacement) involves discrepant long-term results that depend mainly on pre-operative systemic ventricular function; it can be too risky for patients who have undergone previous open-heart surgery and have other comorbidities. Many case reports of patients with CHD and atrioventricular regurgitation repaired percutaneously have been published. This technique seems safe and yields short-term clinical and hemodynamic improvements. The specifics of each patient's complex anatomy must be considered carefully pre-operatively, and more research is needed to assess the long-term outcomes and precise eligibility criteria.

## Abbreviations

acTGA: atrial correction of transposition of the great arteries
ASD: atrial septal defect
AV: atrioventricular
AVVR: atrioventricular valve regurgitation
ccTGA: congenitally corrected transposition of the great arteries
CHD: congenital heart disease
CT: cardiac tomodensitometry
EROA: effective regurgitant orifice area
IAS: interatrial septum
LV: left ventricle
LVEF: left ventricle ejection fraction
MRI: magnetic resonance imaging
MV: mitral valve
NYHA FC: New York Heart Association functional class
RV: right ventricle
RVEF: right ventricle ejection fraction
SLV: systemic left ventricle
SRV: systemic right ventricle
TR: tricuspid regurgitation
TTE: transthoracic echocardiogram
TEE: transesophageal echocardiogram
TV: tricuspid valve
VSD: ventricular septal defect

## 1 Background

Transcatheter valve therapy is the newest frontier in interventional cardiology and is typically performed in older patients with comorbidities. Transcatheter edge-to-edge repair (TEER) for regurgitant tricuspid or mitral valves is an alternative to surgery that has been widely accepted in the last two decades for acquired cardiopathies and is now part of international guidelines [1].

Congenital heart disease (CHD) generally affects younger adults who are at higher risk for surgical complications and have had previous open-heart surgery. Currently, no percutaneous device is approved for treating atrioventricular valve regurgitation (AVVR) in adults with CHD. Few reports have been published on its use mainly in the systemic right ventricle (SRV), and especially in unoperated congenitally corrected transposition of the great arteries (ccTGA) and atrial corrected transposition of the great arteries (acTGA). The majority of these patients develop some degree of SRV dysfunction over the long term. Many confounders contribute to the guarded prognosis of SRV, especially tricuspid regurgitation (TR), which has a complex role in SRV failure as it can be the cause by its intrinsic abnormalities and the consequence [2]. TR has unequivocal effects on functional status, SRV function, and mortality [3]. Medical treatment of SRV and TR is empirical and has not proven effective [4]. Surgery (repair or valve replacement) remains controversial because of discrepancies in reported results and high peri-operative complication rates [5,6]. As a pre-operative condition, RV dysfunction predicts lower survival after surgical interventions [7,8]. Therapies including surgical anatomic correction [9], pulmonary banding [10], and resynchronization [11] might improve AV regurgitation, but data are limited and results tend to be better in the early stages of the disease. Pulmonary hypertension and subpulmonary ventricular dysfunction make heart transplantation and ventricular assist device placement difficult. In CHD with systemic left ventricle (SLV) as well, the high operative risk often related to several previous heart surgeries, make the care team look to less invasive options. In this context, TEER has been considered in recent case reports for such patients and studies are ongoing (see Table 1).

**Table 1:** Published case-reports on percutaneous edge-to-edge repair of *atrioventricular valve* regurgitation in systemic right ventricle.

Reference	CHD	Preoperative imaging data	Devices (position)	Mean gradient (mmhg)	Time of follow-up	Benefits	Adverse events
Franzen and al, Congenital Heart Dis, 2011	ccTGA	RV dysfunction	1 Mitraclip (antero-septal)	n/a	3 months	NYHA FC 3 → 1	0
						6-min walk test 255 → 405 m	
						TR: severe → mild	
Picard and al, 2017	ccTGA	RV MPI 0,51	1 Mitraclip (posteroseptal)	2	2 years	NYHA FC 3 → 2	0
						TR: severe → mild	
						RV MPI 0,47	
Van Melle and al, Neth heart J, 2016	ccTGA with dextrocardia	RV dysfunction	1 Mitraclip (n/a)	2	6 months	Dyspnea improvement	0
						TR: severe → mild	
Gaydos and al, Cardiovasc Revasc Med, 2020	ccTGA	RVEF 28% (CT) Midly Ebsteinoide TV with malcoaptation	1 Mitraclip XTR (n/a)	n/a	6 weeks: 8 months	Hemodynamic: mPAP 35 → 19mmhg; PVR 4,5 → 1,8WU	0
						NYHA FC 3 → 1	
						TR: severe → moderate	
Alshawabkeh and al, Cardiovasc Interv, 2020 case 2	ccTGA	RV FAC 40% (TTE) Central jet due to thethered leaflets	2 Mitraclips XTR (anteroseptal/posteroseptal)	3	10 months	NYHA FC 3 → 1	0
						TR: severe → mild	
						RV FAC 40 → 37%	
Alshawabkeh and al, Cardiovasc Interv, 2020 case 3	ccTGA	RV FAC 28% (TTE) 2 jets between anteroseptal and posteroseptal coaptation planes	2 Mitraclips XTR (anteroseptal/posteroseptal)	5	4 months	NYHA FC 2 → 1	Subclinical SLD
						TR: severe → mild	
Iriart and al, Catheter Cardiovasc Interv, 2021	acTGA (Mustard)	RVEF 38% (MRI) Septal leaflet prolapse and severe annular dilatation	1 Mitraclip XTR (anteroseptal)	2	6 months	NYHA FC 3 → 1	Common flutter (day 10)
						SF36 PCS score: + 15	
						SF36 MCS score + 10	
						End-diastolic RV volume: 235 → 155 mL/m^2^	
						RVEF: 38%→ 57%	
Tan and al, Catheter Cardiovasc Interv, 2020	Heterotaxy syndrome, unbalanced AV canal defect, DORV, palliated with bidirectional Glenn shunts	TAPSE 1.3 cm dp/dt 500mmhg/s Functional AVVR secondary to annular dilation	3 Mitraclips XTR (between anterior and posterior bridging leaflets)	1–2	12 weeks	Symptoms improvment TAPSE 1.2 cm, dP/dt 625 mmHg/s	0
Blusztein and al, JACC Case report, 2022	HLHS palliated with extracardiac Fontan procedure	RV dysfunction Functional central TR	3 Mitraclips XTW (anteroseptal, anteroporerior, posteroseptal)	3	5 days		Death
Luedike and al, JACC, 2021	ccTGA	RVEF 33% (MRI)	1 Pascal	1	4 days	NYHA FC improvement BNP: 2479 → 1587 pg/mL TR: torrential → trace	0
Lewis and Al, Eur Heart J Case Rep, 2021	ccTGA	RVEF 32%	2 Mitraclips XTR	n/a	7 months	NYHA FC: 3 → 1	Subclinical posterior SLD
		LVEF 59% (MRI)					
		Prolapse of A2 segment					

*acTGA: atrial corrected transposition of the great arteries; AVVR: atrioventricular regurgitaton,; CT: cardiac tomodensitometry; ccTGA congenitally corrected transposition of the great arteries; CHD: congenital heart disease; ODRV: double outlet right ventricle; EF: ejection fraction; FAC: fractional area change; HLHS: hypoplastic left heart syndrome; mPAP: mean pulmonary arterial pressure; MPI: myocardial performance index; NYHA FC: New York Heart Association Functional Class; PVR: pulmonary vascular resistances; RV: right ventricle; MCS: mental component summary score; PCS/physical component summary score; SLD: single leaflet detachment; TTE: transthoraic echocardiography; TR: tricuspid regurgitation; TV: tricuspid valve*.

## 2 Methods

We searched PubMed, Google Scholar, Science direct for all English-language case-reports and series of patients with congenital heart disease (CHD) and atrioventricular regurgitation, treated with transcatheter edge-to-edge repair, using the following keywords: Mitraclip, edge-to-edge repair, congenital heart disease. All CHD (including systemic right ventricle and systemic left ventricle) were reported. This review outlines the anatomical and technical challenges related to the type of CHD, the benefits and the adverse events in the follow-up (see Table 2).

**Table 2:** Published case-reports on percutaneous edge-to-edge repair of atrioventricular valve regurgitation in systemic left ventricle.

Reference	CHD	Preoperative imaging data	Device (position)	Mean gradient (mmhg)	Time of follow-up	Benefits	Adverse events
Alshawabkeh and al, Cardiovasc Interv, 2020 case 1	Dextrocardia, DORV, severe pulmonary stenosis large ASD, palliated with percutaneous Glenn anastomosis	LVEF 60% (TTE) Prolapse of mitral A2 segment	1 Mitraclip XTR (A2 P2)	1	4 months	NYHA FC 3 → 2	0
						TR: severe → mild	
						LVEF 60 → 67%	
Willemsem and al, Eur Heart J, 2014	Isolated mitral cleft	LVEF 15% (TTE)	2 Mitraclips (posteromedial and anterolateral along the cleft)	n/a	n/a	n/a	0
Russo and al, JACC Case Rep, 2020	Mitral cleft and primum AV canal defect previously repaired	LVEF 40% (TTE)	2 Mitraclips NTR (medial to the cleft and between A1 and A2)	n/a	n/a	n/a	0
Stys and al, Euroitervention, 2019	MV cleft and AV septal defect previously repaired	LVEF 50–55% (TTE)	2 Mitraclips (A1-P1 and A3-P2)	n/a	1 month	Symptoms improvement	0
						TR: severe → mild	
Gorenflo and al, Cardiology in the young, 2013	Severe aortic valve stenosis and dysplasia of the mitral valve	n/a	1 Mitraclip (A2 P2)	n/a	2 weeks	NYHA FC 4 → 2	0

AVVR: atrioventricular regurgitaton,; CHD: congenital heart disease; DORV: double outlet right ventricle; EF: ejection fraction; LV: left ventricle; MR: mitral regurgitation; NYHA FC: New York Heart Association Functional Class; TTE: transthoraic echocardiography.

## 3 TEER for treating AVVR in systemic right ventricle

TEER has been used to treat severe systemic TR in a few patients deemed at high surgical risk because of previous cardiac surgeries or other comorbidities.

The first case report in 2011 [12] involved a symptomatic 62-year-old woman diagnosed with ccTGA with severe TR and SRV dilation and dysfunction. As she refused surgery, a MitraClip was implanted between the septal and anterior leaflets via transseptal access. Three months later, her functional status had improved from New York Heart Association functional class (NYHA FC) 3 to 1, as had her 6-min walking test.

Picard et al. reported a similar patient with ccTGA with severe TR, SRV dilation, and mild systolic dysfunction hospitalized for heart failure [13]. At the 2-year follow-up, the authors reported clinical improvement without hospitalization for heart failure, and transthoracic echocardiography (TTE) revealed moderate TR and unchanged SRV volume and function.

Alshawabkeh et al. [14] reported three patients with CHD undergoing TEER, including a 75-year-old woman with native ccTGA who developed symptomatic progressive functional TR. Two MitraClip XTR were deployed between the anterior and septal leaflets, and between the anterior and posterior leaflets, despite a small challenging posterior leaflet. The authors reported an immediate reduction in regurgitation with near normalization of the pulmonary vein flow. Subsequently, a subclinical single leaflet detachment (SLD) of the anteroposterior clip caused mild to moderate TR. No further intervention was performed, and at the 10-month follow-up the patient's NYHA FC had improved from class 3 to 1 and her regurgitation severity was unchanged. Another patient with ccTGA in this series had MitraClip XTR implanted between the posterior and septal leaflets, and between the anterior and septal leaflets and exhibited clinical and echocardiographic improvement at the 4-month follow-up.

Van Melle et al. reported the first case of ccTGA with dextrocardia in 2016 [15]. Six months after valve clipping, the patient's TR was mild and heart failure symptoms were reduced.

The hemodynamics of TEER in SRV were illustrated in a case involving a 45-year-old woman with ccTGA who presented with severe systemic TR, biventricular systolic failure and pulmonary hypertension [16]. Invasive left atrial monitoring revealed an immediate improvement from 37 to 22 mmHg after one MitraClip XTR. At the 8-month follow-up, the patient had significant symptomatic improvement, while TTE revealed moderate systemic TR and anterograde pulmonary venous flow on Doppler. The subpulmonary LV function was improved, but the SRV dysfunction remained unchanged. Six weeks after the procedure, diagnostic catheterization revealed a normal mPAp of 19 mmHg and PVR of 1.8 WU, while repeat catheterization at 7 months revealed mild elevation of the mPAp to 27 mmHg, but a normal PVR of 2 WU.

Previously, our institute reported the first case of acTGA with severe TR treated percutaneously [17]. This 48-year-old patient had undergone a Mustard operation as an infant and was admitted with severe TR with septal leaflet prolapse and tricuspid annular dilation. He was treated with a MitraClip XTR in the antero-septal commissure using transbaffle access. He was discharged 2 days after the procedure, but returned 7 days later with atrial flutter, which was ablated through an additional transbaffle puncture just below the atrial septal defect (ASD). Assessments at 1 and 6 months revealed marked clinical improvement (NYHA FC 3 to 2), stable TR on TTE, reduced end-diastolic SRV volumes (from 235 to 155 mL/m^2^), and increased RVEF (from 38% to 57%) on magnetic resonance imaging (MRI).

Tan et al. reported a unique case of TEER repair using a MitraClip in a single-ventricle patient with severe common AVVR and single-ventricle systolic dysfunction [18]. The patient was a 35-year-old man born with a double outlet right ventricle and an unbalanced atrioventricular canal defect. He was treated with bilateral bidirectional Glenn shunts, but not Fontan palliation due to an elevated single-ventricle end-diastolic pressure. At his 12-week follow-up, he noted significantly improved stamina; his oxygen saturation was improved from before the procedure; and TTE revealed moderate residual AVVR, while the single ventricular function was stable.

In 2022, Blusztein and al reported the first TEER repair of a systemic tricuspid valve (TV) in an extracardiac Fontan patient born with hypoplastic left heart syndrome (HLHS), presenting with cardiogenic shock [19]. They accessed via the right internal jugular vein because of concerns of the lack of “height” using an inferior vena cava approach. TR was reduced from torrential to 2+ moderate but unfortunately the patient died 5 days later of multiorgan failure.

Recently, the first case of TEER performed with the Pascal system was reported in a patient with ccTGA and systemic TR [20] (see Table 1) (see Table 2).

Finally, one paper reported the first case of a subpulmonary right-sided mitral regurgitation (MR) due to anterior prolapse in a patient with ccTGA, using 2 Mitraclips XTR, the second one resulting in posterior single leaflet detachment [21]..

## 4 TEER for treating AVVR in systemic left ventricle

The MitraClip has been used to treat MR in congenial heart disease with systemic left ventricle.

Alshawabkeh et al. treated a 39-year-old woman with a double-outlet severely hypoplastic RV, mildly hypoplastic TV, large ventricular septal and secundum atrial septal defects (ASD), who underwent percutaneous cavopulmonary anastomosis at the age of 25 years [14]. She was diagnosed with severe MR due to A2 prolapse. Puncturing the interatrial septum (IAS) was necessary despite the large ASD to provide optimal support for the guide catheter, but that was challenging due to the abnormal angle between the IAS and mitral valve plane. A MitraClip XTR was successfully implanted in the A2-P2 position and decreased the MR. The patient's systemic oxygen saturation and symptoms had improved at the 4-month follow-up.

Willemsen et al. reported a case involving a 51-year-old patient with a mitral posterior leaflet cleft who underwent successful implantation of two close clips symmetrically on each side of the cleft, perpendicular to the mitral closure orifice [22]. The patient's MR was markedly reduced and no stenosis was observed.

Another case involved a 67-year-old woman with congenital mitral cleft and a primum ASD repaired 40 years previously, but with a recent diagnosis of severe MR due to a thickened mid-A2 segment and a cleft between A2 and A1 with a distorted anterior mitral leaflet [23]. Two Mitraclips NTR were implanted, with mild residual MR and significant improvement in the pulmonary venous flow. Generally, a second clip is implanted as parallel as possible lateral to the first; in this case, however, the cleft limited the grasp immediately adjacent to the first clip, so the second MitraClip was implanted so that it grasped the cleft scallops (perpendicular to the first clip), which is similar to the surgical repair.

Stys et al. reported a female patient with a history of MV cleft repair and ASD repair for a partial AV canal defect with a pericardial patch and subsequent AMPLATZER Septal Occluder device [24]. She presented with severe MR due to cleft recurrence in the A2 position. Due to the IAS particularities, the transseptal puncture was performed in the posteroinferior segment, rather than the classic posterosuperior segment. Initially, a MitraClip was deployed in a lateral position at the A1-P1 scallop, but it did not reduce the MR. Due to the A2 cleft, they placed a second MitraClip at a slight angle to the first at the A3-P2 scallop in a “V” configuration. At the 1-month follow-up, the MR was classified as mild and the patient reported improved symptoms.

The first child to undergo a Mitraclip repair with clinical improvement 2 weeks later was an adolescent with recurrent aortic stenosis needing 3 surgeries and mitral dysplasia with severe MR at the age of 15 [25].

This review don't take into account “pseudo-clefts” on the posterior leaflet tip due to asymmetric left ventricular dilatation in non congenital heart disease.

## 5 Discussion

Transcatheter edge-to-edge repair in CHD appears to be an efficient short-term option for patients deemed ineligible for surgical valve repair or replacement. Most of the cases reported indeed a significant improvement in heart failure symptoms, mainly dyspnea. Iriart an Al [17] even reported better quality of life scores after 6 months. Despite the difficulty to quantify regurgitation in complex CHD, most of the procedures have permitted to decrease the TR or MR severity from severe to mild or moderate. There was not deterioration in systemic ventricular function and occasionally, improvement was reported [17].

No intraprocedural death has been reported. One death due to multiorgan failure was reported 5 days after the procedure in a patient admitted with cardiogenic shock. One patient with acTGA developed flutter early after the procedure, but he already had a history of flutter [17]. Two papers reported a posterior SLD without clinical consequence [14,21]. No reports of valvular stenosis (mean gradient >5 mmHg) have been published. In comparison, the MITRA-FR trial assessing MitraClip in functional acquired MR did not report any periprocedural deaths and the rate of periprocedural complications was 14.6%, including procedural failure (4.2%), hemorrhage needing transfusion (3.5%), significant IAS lesions (2.8%), cardiogenic shock (2.8%), embolism (1.4%), and tamponade (1,4%) [26]. In the TRILUMINATE trial assessing TriClip in acquired TR, the rate of major adverse effects at 1 year was 7.1% and five cases of SLD were observed without clinical consequences or TR worsening and with no new cases occurring after 30 days [27]. Four subjects had mean tricuspid valve gradients >5 mmHg at the 1-year follow-up with no related clinical symptoms and no further intervention was required.

It is difficult to extrapolate these procedures from acquired cardiopathies to CHD given the huge anatomical differences. First, valve access requires individual assessment, including CT, MRI, transesophageal echocardiography, fluoroscopy, and per-procedure fusion imaging. We previously described the different procedural steps in an acTGA patient [17]. With regard to ccTGA, Franzen et al. reported the procedural differences for acquired MR TEER and emphasized the need for a higher position 3.8–4 cm from the mitral annulus when performing the transseptal puncture, a more medial position during the steering maneuver, and the orientation angle for clip positioning [12]. The etiology of the AV regurgitation also determines the procedural strategy, as demonstrated by the approach needed for a mitral cleft [22,23]. Besides, septal lesions from prior surgery have to be known when performing *trans*-septal puncture. Furthermore, the chance of successfully grasping the leaflets needs to be assessed. Criteria outlining the suitable anatomy for mitral valve repair have now been established for non-congenital patients, and include central MR, non-rheumatic valve, coaptation length >2 mm, coaptation depth <11 mm, no calcification, MV area >4 cm^2^, flail >15 mm, and flail gap >10 mm [28]. No such unequivocal criteria have been established for subpulmonary tricuspid repair.

It is also difficult to predict which patients will benefit most from TEER. Grayburn et al. [29] proposed the concept of proportionate and disproportionate MR as to explain the discrepancy between the COAPT trial [30], which reported fewer hospitalizations for heart failure at 2 years in the MitraClip group versus medical therapy and the MITRAFR trial [26], which did not report differences in the primary composite end points in the two groups (death or hospitalization for heart failure). In MITRAFR, MR proportionate to the effective regurgitant orifice area (EROA) was as high as expected with the LV volume, while in COAPT, EROA was 30% higher and the LV volumes 30% lower. More research will be required to assess whether this concept is applicable to CHD.

Almost all cases described above were performed using MitraClip XTR or NTR, which are third-generation MitraClip devices, characterized by clip delivery shaft improvements, the ability to open and close clip arms without locking the device, and longer clip arms on the XTR. Since 2019, fourth-generation devices have been used; these are characterized by wider clip arms, four different clip sizes (NT, NTW, XT, and XTW), and controlled gripper actuation. The choice of device depends partly on the anatomy of the valve, including leaflet length, and this requires preoperative assessment. Other TEER devices are used worldwide in acquired cardiopathies, and include the TriClip device (Abbott) with a differentiated delivery system adapted to RV or the Pascal device (Edwards Lifesciences). Luedike et al. implanted the first Pascal device in ccTGA and reported that the combination of the central spacer coupled with broad, contoured paddles reduces leaflet stress, which is thought to translate into reduced pressure gradients across the valve [20]. However, it is not clear whether one technique is superior to the others when treating these complex anatomies.

The major limitation of this review relates to the nature of the papers (case-reports) which of course induces a biased selection of successful procedures. This is due to the recentness of such procedures in those rare congenital diseases. More systematic studies are needed to generalize results. Moreover and for the same reasons, the reported short-term follow-ups (generally few months) prevent from extrapolating to long-term benefits.

## 6 Conclusion

Percutaneous edge-to-edge repair appears to be a safe, effective therapeutic option for atrioventricular regurgitation in patients with CHD who are at high risk for surgical complications. Studies to assess the long-term clinical and hemodynamic improvements are ongoing.

## Sources of funding

No funding was provided.

## Declaration of competing interest

The authors declare that they have no known competing financial interests or personal relationships that could have appeared to influence the work reported in this paper.
